# Editorial to “Carbon dioxide insufflation to facilitate epicardial access in extracorporeal membrane oxygenation‐supported ventricular tachycardia ablation”: Blowing an exhaled gas for easy and safe pericardial puncture

**DOI:** 10.1002/joa3.13216

**Published:** 2025-01-14

**Authors:** Ugur Canpolat

**Affiliations:** ^1^ Arrhythmia and Electrophysiology Unit, Department of Cardiology Hacettepe University Faculty of Medicine Ankara Turkey

**Keywords:** carbon dioxide insufflation, epicardial catheter ablation, ventricular tachycardia

In the current issue of the *Journal of Arrhythmia*, Takase et al.^1^ reported a challenging patient with scleroderma‐related structural heart disease who was admitted with recurrent ventricular tachycardia (VT) episodes after a failed endocardial catheter ablation alone. The authors' first challenge during an index catheter ablation was the hemodynamic instability during VT for mapping. The author's second challenge during the planned second catheter ablation was the anatomical neighboring of the left hepatic lobe to the subxiphoid epicardial access route. The authors have overcome both challenges with the carbon dioxide (CO_2_) insufflation method for facilitating the visibility of intrapericardial space and the hemodynamic support of extracorporeal membrane oxygenation. The endocardial and epicardial catheter ablation was successfully performed by overcoming these technical obstacles.

Catheter ablation is advised to reduce recurrent VT and the need for implantable cardioverter defibrillator shocks in patients with non‐ischemic cardiomyopathy (NICM) and recurrent sustained monomorphic VT when antiarrhythmic medications are ineffective, contraindicated, or poorly tolerated.^2^ VT developed as a result of left or right ventricular myocardial involvement, and successful catheter ablation has been previously reported in patients with systemic scleroderma.^3^ However, due to the underlying mechanism of myocardial disease and VT (primarily caused by scar‐related reentry^3^), endocardial catheter ablation alone might be insufficient to eliminate the VT focus. Furthermore, the contribution of ventricular scar to the electrophysiological abnormalities targeted for endocardial ablation of unstable VT differs between ischemic and non‐ischemic cardiomyopathies. Since the case of Takase et al. also involved VT due to non‐ischemic etiology, endocardial substrate ablation alone may have failed for hemodynamically unstable VT. Epicardial catheter ablation of VT can be useful after the failure of endocardial ablation because of the higher rate of the intramyocardial and epicardial substrate in patients with NICM.^2^ Demonstrating a three‐dimensional hyperboloid VT circuit structure is another reason that endocardial catheter ablation alone is ineffective in some patients.^4^ Before epicardial catheter ablation, pre‐procedural imaging techniques, such as cardiac computed tomography or magnetic resonance imaging, may play a critical role in procedural guidance and preventing complications by indicating neighboring structures.^2^ Accessing the epicardium is typically achieved through a subxiphoid and trans pericardial puncture. However, epicardial access may be difficult due to anatomical obstacles and poor fluoroscopic visibility, which result in both acute and delayed complications. Carbon dioxide insufflation through the coronary sinus exit is a novel approach to address the technical challenges of epicardial access, especially in patients with minimal or no pericardial fluid (dry epicardium), and adhesions (previous history of cardiovascular surgery, epicardial catheter ablation, or pericarditis). The technique is safe and effective, minimizing the risks associated with conventional approaches.^5^ It also delineates the localized pericardial adhesions in high‐risk patients and guides for target epicardial access sites.^6^ This technique may show that epicardial access is impossible in some patients with advanced pericardial adhesions.^6^ Takase et al.^1^ noted that an important step in the procedure is to avoid perforating the coronary veins that run toward the left ventricular muscle. Observing premature ventricular contractions after advancing the wire into those branches and staining of the myocardium following contrast injection are key findings indicating inappropriate coronary vein perforation. Chaumont et al.^7^ reported similar success rates of epicardial access via conventional versus CO_2_ insufflation method. However, the CO_2_ insufflation method was significantly linked to lower rates of major complications and bleeding compared to conventional access. This multicenter study illustrates that the primary limitation of distal coronary venous exit for CO_2_ insufflation is the lengthy nature of the technique, which involves several laborious steps. In addition to its well‐known efficacy and safety profile, the technique can be performed easily and successfully in both low‐ and high‐volume centers. Additionally, this technique displaces the diaphragm downward due to the carbon dioxide injected into the pericardial space, pushing the left lobe of the liver away from the epicardial access route.^1^ In conclusion, although the CO_2_ insufflation technique involves time‐consuming steps, it is a safe and effective method for achieving epicardial access in patients undergoing VT catheter ablation. This technique, which demonstrates pericardial adhesions before puncture, should be considered, especially in high‐risk patients requiring epicardial access.

## CONFLICT OF INTEREST STATEMENT

Authors declare no conflict of interests for this article.

## References

[1] Takase T , Yoshikawa K , Morimoto T , Kamiya K , Furukawa Y . Carbon dioxide insufflation to facilitate epicardial access in ECMO‐supported ventricular tachycardia ablation. J Arrhythm. 2025:45: e13188. 10.1002/joa3.13188 PMC11730983 39817015

[2] Cronin EM , Bogun FM , Maury P , Peichl P , Chen M , Namboodiri N , et al. 2019 HRS/EHRA/APHRS/LAHRS expert consensus statement on catheter ablation of ventricular arrhythmias. J Arrhythm. 2019;35(3):323–484. 31293696 10.1002/joa3.12185 PMC6595359

[3] Rankin AC , Osswald S , McGovern BA , Ruskin JN , Garan H . Mechanism of sustained monomorphic ventricular tachycardia in systemic sclerosis. Am J Cardiol. 1999;83(4):633–636. 10073883 10.1016/s0002-9149(98)00935-7

[4] Nishimura T , Shatz N , Weiss JP , Zawaneh M , Bai R , Beaser AD , et al. Identification of human ventricular tachycardia demarcated by fixed lines of conduction block in a 3‐dimensional hyperboloid circuit. Circulation. 2023;148(18):1354–1367. 37638389 10.1161/CIRCULATIONAHA.123.065525

[5] Cerantola M , Santangeli P . Epicardial access facilitated by carbon dioxide insufflation via intentional coronary vein exit: step‐by‐step description of the technique and review of the literature. J Interv Card Electrophysiol. 2023;66(1):109–116. 35963909 10.1007/s10840-022-01338-2

[6] Chaumont C , Oraii A , Markman TM , Garcia FC , Lin D , Supple GE , et al. Epicardial carbon dioxide insufflation is a novel technique for the identification of epicardial adhesions and targeting epicardial access. Heart Rhythm. 2024;21(7):1040–1041. 38280620 10.1016/j.hrthm.2024.01.034

[7] Chaumont C , Oraii A , Garcia FC , Supple GE , Santangeli P , Kumareswaran R , et al. The safety and efficacy of epicardial carbon dioxide insufflation compared with conventional epicardial access. JACC Clin Electrophysiol. 2024;10(7 Pt 2):1565–1573. 38864808 10.1016/j.jacep.2024.05.004

